# Abrocitinib for pediatric Toxicodendron contact dermatitis

**DOI:** 10.1016/j.jdcr.2026.07.033

**Published:** 2026-07-25

**Authors:** Liyang Duan, Xianjie Wu

**Affiliations:** Department of Dermatology, Second Affiliated Hospital, Zhejiang University School of Medicine, Hangzhou, China

**Keywords:** abrocitinib, allergic contact dermatitis, Janus kinase inhibitors, pediatric, Toxicodendron dermatitis

## Introduction

Allergic contact dermatitis (ACD) is a delayed-type hypersensitivity reaction mediated by T cells in response to external allergens.^1^ Standard management including prevention and symptomatic treatment with topical corticosteroids, calamine lotion, and oral antihistamines has limitations, such as steroid risks in children and refractory cases.^1^^,^^2^

## Case presentation

**Fig 1:**
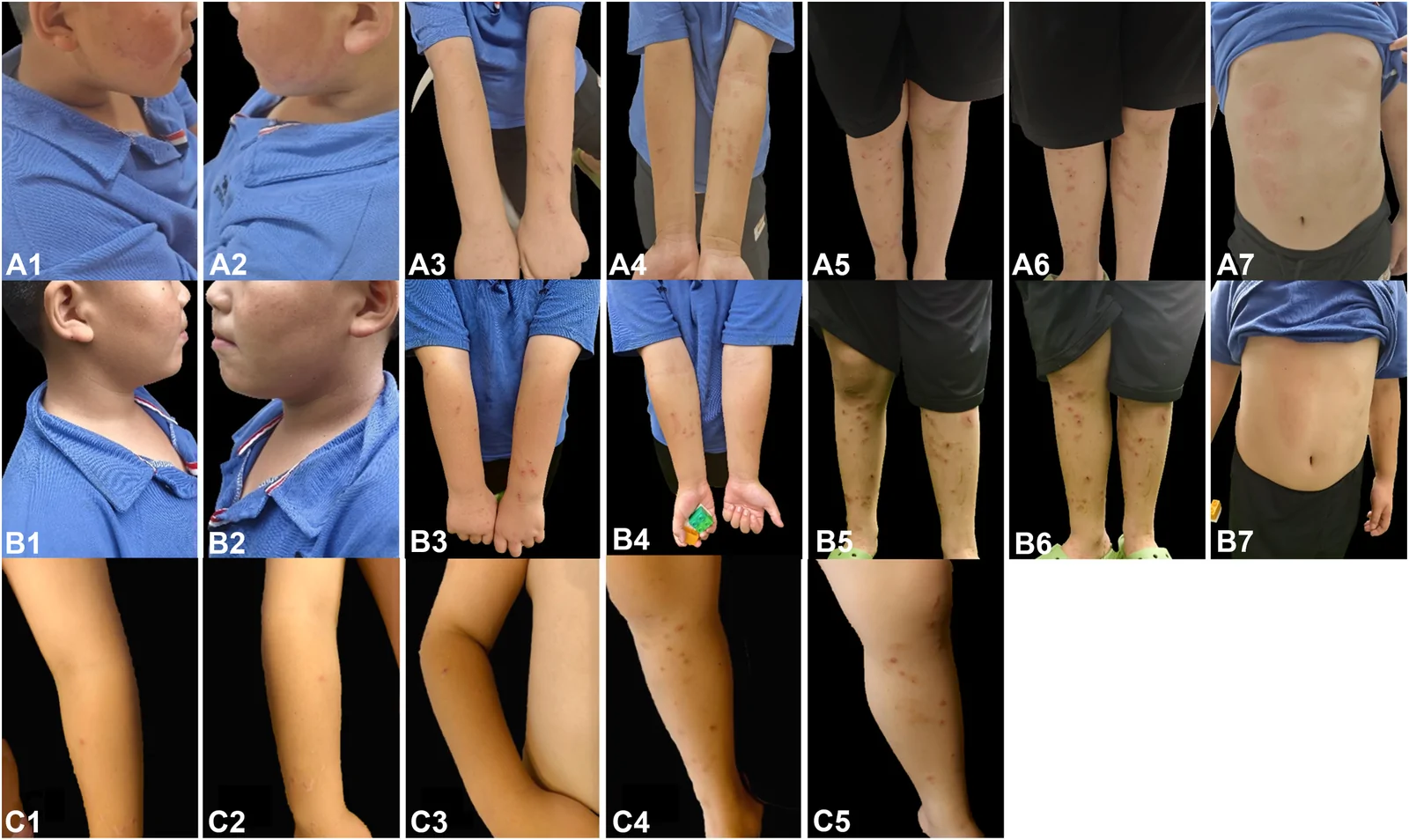
**A,** Day 0 of abrocitinib treatment, facial erythema with diffuse erythematous papules and plaques on the trunk and limbs. **B,** Day 3 of abrocitinib treatment, facial erythema has resolved; papules and plaques on the trunk and limbs have significantly improved. **C,** Day 11 of abrocitinib treatment, only mild post-inflammatory hyperpigmentation remains.

An 8-year-old boy (weighing 44 kg) presented with severe, intensely pruritic erythematous papules and plaques involving the trunk and limbs for 10 days following lacquer tree (Toxicodendron vernicifluum) exposure. Despite 3 days of conventional therapy for the initially diagnosed eczema/allergic dermatitis, which included topical hydrocortisone, emollients (vitamin E/urea), and oral levocetirizine, the dermatitis worsened and extended to the face, thus being deemed refractory to initial topical therapy. SCORAD (Scoring Atopic Dermatitis) index^3^ was 56.1, indicating severe disease (Fig 1, *A*).

The diagnosis was revised to Toxicodendron contact dermatitis. His past medical history was unremarkable. Informed consent was obtained from the patient's parent for both the treatment and the publication of this case report. A short course of systemic corticosteroids, the standard first-line systemic therapy for moderate-to-severe cases, was recommended but explicitly declined by the parents due to concerns regarding potential adverse effects on their child's growth and development. Conventional therapy was discontinued, and after thorough counseling and shared decision-making, treatment was initiated with oral abrocitinib (50 mg once daily) and cetirizine (10 mg nightly). This dose was selected based on recent real-world evidence supporting the safety and efficacy of 50 mg daily in children aged 6 to 11 years with moderate to severe atopic dermatitis, and was tailored to the patient’s age and severity of disease.^4^

After 3 days of treatment, the patient's pruritus resolved. Facial erythema and edema cleared completely, and lesions on the trunk and limbs showed marked improvement (SCORAD 28) (Fig 1, *B*). By day 11, near-complete clinical resolution was achieved, with only minor residual erythema and hyperpigmentation on the legs (SCORAD 18.4) (Fig 1, *C*).

## Discussion

Allergic contact dermatitis (ACD) induced by Toxicodendron genus is the most prevalent plant-related dermatitis in the United States.^5^ The allergen urushiol triggers a T cell-mediated immune response. Effector CD4+ T cells orchestrate the release of proinflammatory cytokines (IFN-γ, IL-2, IL-17, IL-21, IL-22), while CD8+ T cells directly contribute to cytotoxicity and inflammatory amplification.^1^ In sensitized individuals, characteristic linear, pruritic eruptions typically develop within 24 to 48 hours, progressing from erythema and papules to vesicles or bullae.

Standard management includes topical corticosteroids, calamine lotion, cool compresses, and oral antihistamines. Moderate-to-severe cases may require systemic corticosteroids.^1^^,^^2^ However, systemic corticosteroids are not always desirable in children due to potential side effects. Even short-course oral corticosteroids in children have been associated with transient growth suppression, weight gain, behavioral and mood disturbances, hyperglycemia, and hypothalamic-pituitary-adrenal axis suppression.^6^^,^^7^ In the present case, the parents declined systemic corticosteroids specifically because of these concerns. Additionally, some cases are refractory to conventional therapy. In this case, the patient's dermatitis progressed to severe intensity (SCORAD index 56.1) including facial involvement despite initial appropriate therapy.

Abrocitinib, a selective JAK1 inhibitor approved for moderate-to-severe atopic dermatitis (AD), offers potent anti-inflammatory and antipruritic effects.^8^ The rationale for selecting abrocitinib in this case was multifactorial. First, allergic contact dermatitis is driven by effector T cells that release proinflammatory cytokines such as IL-4, IL-13, IL-31, IL-22, and IFN-γ, many of which signal through the JAK-STAT pathway with JAK1 playing a central role. Abrocitinib directly blocks the intracellular signaling of these key mediators, thereby simultaneously suppressing inflammation and the hallmark pruritus of ACD.^9^^,^^10^ Second, unlike corticosteroids, abrocitinib is not associated with growth suppression or adrenal insufficiency, as it does not act on the hypothalamic-pituitary-adrenal axis. Limited real-world evidence in children aged 6 to 11 years with moderate-to-severe atopic dermatitis, including the use of abrocitinib 50 mg once daily, provided a clinical reference for dose selection and risk assessment in this age group.^4^ Third, the patient's rapidly worsening condition (SCORAD 56.1) with facial involvement necessitated prompt systemic intervention; delaying treatment risked further deterioration, skin barrier disruption, and secondary infection. In this case, abrocitinib led to a dramatic and rapid response, with pruritus resolving within 3 days and near-complete clearance achieved by day 11. To our knowledge, this is the first reported case demonstrating the successful and safe use of abrocitinib in a child under 12 for Toxicodendron contact dermatitis.

While systemic corticosteroids remain a standard option for severe ACD, JAK inhibitors may offer a targeted alternative without the typical corticosteroid side-effect profile, though their own specific risks require consideration. Notably, the term “refractory” in this case denotes refractoriness to optimized topical treatment, not to systemic corticosteroids, as the latter were declined by the parents. This represents a limitation of our report, as we cannot demonstrate refractoriness to standard systemic therapy.

In conclusion, this case suggests that oral abrocitinib can be a highly effective and well-tolerated treatment for severe, topical-therapy-refractory Toxicodendron contact dermatitis in pediatric patients, particularly when systemic corticosteroids are refused by the family, highlighting a novel off-label application worthy of further investigation.

## Conflicts of interest

None disclosed.
